# Remarks upon Catalepsy, with Report of a Case

**Published:** 1875-01-15

**Authors:** J. H. Hollister


### REMARKS UPON CATALEPSY, WITH REPORT OF A CASE.

Submitted to the Chicago Society of Physicians and Surgeons, by J. H. Hollister, M. D.

GENTLEMEN.—The affection termed Catalepsy, is one so exceedingly rare, and in many of its phenomena so interesting that I deem it hardly necessary to apologize for calling your attention to the subject, and for occupying a few moments in detailing a case which has fallen under my observation, which so far as I am able thus far to study it, *seems* one of genuine catalepsy. This opinion has the concurrence without exception, so far as I know, of a number of medical men, who have been interested in examining the patient, with me, since he entered the hospital.

Few men, even among our medical writers, seem to have much experience from personal observation; some even doubting, but that, among many cases of so-called catalepsy, they had not all been feigned, or were a peculiar manifestation of *Hysteria*. These suggestions have led me from day to day to watch the case all the more critically, lest I should be deceived as to the *facts*.

Catalepsy is indeed so rare, that even Prof. Watson, with all his extended facilities for observation, remarks, "I have never seen a case of perfect catalepsy—which I now regret, as I once had an opportunity of doing so, of which I did not avail myself,"—and in his work, cites quite at length, a case which is quite fully detailed by Prof. Gooch.

Prof. Niemeyer says, "In my description of the symptoms and course of catalepsy as an independent disease, I must rely entirely upon the representations of others, since all the cases which I have had an opportunity of observing personally have inspired me with the suspicion that they were simulated." Prof. Dunglison in like manner, refers to its rare occurrence, and speaks of a single case in which it had occasional very brief manifestations which had fallen under his observation.

It will be my purpose in few words to give a portraiture of the characteristic symptoms of catalepsy, and with this compare the appearance and symptoms of my patient as they have been noted from day to day, and I shall ask you gentlemen to sit in judgment upon the question of its genuineness.

Blazius classes catalepsy with the neuroses of stability. During the *fit*, the limbs remain stationary in the position assumed before the attack, or in that in which the attendant has placed them, and are not susceptible of change so far as can be judged by the will of the patient. In a quiescent state, the muscles do not seem in violent contraction, in fact, slight motion is easily accomplished *but* only to a very limited extent, for the moment anything like free movement of any portion of the body is attempted, the rigidity of the muscles is very like that of a person when endeavoring voluntarily to oppose the motion you seek to make, only more *steady*, and for almost any length of time persistent, without the tremor which invariably appears when the muscles are for a long time upon severe strain in obedience to the will.

Besides the loss of voluntary motion, there is also a suspension of both *thought* and sensibility. If not, if the consciousness and sensibility be retained, there is no power by word or sign to indicate their continuance.

There are instances of partial catalepsy, when motion and sensibility were *lost*, but consciousness was not suspended; but in cases of typical catalepsy, the patient upon recovery has had no consciousness of sensations nor position. And all these strange phenomena while the functions of organic life were either not at all or but slightly interfered with.

That sensibility is lost, seems evident in that there are not the manifestations of pain which find even involuntary expression when there is pinching, pricking, cutting or burning of the surface of the *body*. The hearing seems lost, and the pupil of the eye responds to the stimulus of light.

The duration of the attacks are often for a few moments, and are sometimes induced by what is termed animal magnetism. Sometimes they are present for a few hours, and then pass away; in cases more rare they are persistent for days.

In respect to duration of attack, the case I am now to relate exceeds any of which I find record.

With reference to the causes which produce catalepsy, a word should be said as this has a bearing upon the case before us. Unusual impressibility of the nervous system, from whatever cause, seems to be the predisposing condition, and it is most frequently complicated with hysteria. It is the sequel of marked inebriety and of insanity. Such are the conditions in which, under the excitement in a powerful manner of the emotions, the person passes into this strangely unconscious state.

I will now proceed to detail the more obvious symptoms and conditions of my patient, reserving for a future paper such observations upon the nervous state as the limits of this article will not permit.

CASE—Michael Finnigan, a laborer, a native of Ireland, 28 years of age, 5 feet 10 inches in height, 38 inches in chest measurement, of good muscular development, but rather spare of habit, weighing about 140 pounds, with fair skin, dark hair and whiskers, and of well-developed nervous temperament, was admitted to Mercy Hospital, Nov. 4, 1874.

We glean from imperfect sources the following brief history: He had been a healthy, hard-working man. For eight years he had drank liquor to excess. During the last year he had left it off, using in place large quantities of very strong tea.

It is said that he left off work in a slaughter-house, a year ago, on account of disordered nerves.

His mother says, that at times he behaved himself very strangely, and was, as I think, in some measure insane. Some eight weeks before his admission, he became in some degree unconscious, but did not fall, recovered himself so far as to reach the house of an acquaintance, with difficulty; became more and more helpless; was remembered to have answered but a single question during that evening, and now so far as I can learn, for a period of nearly *three months*, has been entirely speechless. The condition of the patient from day to day has been noted by my assistant, Dr. H. Gradle, to whose notes I make reference, the statements of which are verified by my own daily observation.

The patient's condition at the time of entrance was that of pretty good health, there being but little appearance of emaciation. Skin warm, moist, pliable, and frequently slightly bathed with perspiration. The temperature per rectum, 99.8. Pulse varying from 65 to 78. Heart sounds nearly natural, but rather feeble. Pulse full; soft; easily compressible. Respirations almost entirely abdominal, and 13 per minute. Mouth firmly closed, and facial muscles fixed and rigid. Eyes closed for hours; occasionally a tear coming over the face from the external canthus—a quick blow upon the nose or forehead would produce the semblance of winking. Globe of the eye mostly turned inward and upward, remaining fixed for long periods of time, the pupil *contracting* somewhat under powerful stimulation. The muscular system seems rigid in every part, save in the motion of the *eye-lids*, and these seem never under the control of volition. The muscles at first yield slightly under pressure, and then powerfully resist farther movement. With considerable force, say of twenty or twenty-five pounds pressure, the upper extremities may be flexed in any direction, in which they are susceptible of motion, and will then remain as placed for almost any period of time—even for hours; and an equal force would be required to return them to their former position. The body can be suspended in nearly a straight line for quite a period of time, by lifting the feet and occiput. The lower extremities raised to an angle of forty-five degrees from the bed, require from eight to ten minutes to gravitate to the level again. The muscles of the mouth are only overcome with much force; and when relaxed, the mouth remains fixed and open for a time. Buccal secretions are natural. Tongue moist and natural. Mucous membranes indicate perfect aeration of the blood and normal secretions. No methods devised by heat, tickling the fauces, inhalation of vapors, currents of electricity, or stimulating or irritating applications, gave us any evidence of consciousness. Such was the state of the patient on the 4th instant, when he was brought to the hospital.

Nov. 23. The patient has now been under my care for nineteen days, and during that time, I have been able to note or learn from the interne, the following facts:

1st. *As to the motions of the body.*—Usually, once in twenty-four hours, he will turn upon his side in bed, and has twice placed his hand under his head. Slight motions of the extremities may be accomplished at will by the attendant, but any decided flexion or extension is always accomplished with difficulty. When the rigidity is overcome by *force*, the part remains as firmly fixed in the position to which it is compelled, as in that from which it was removed. If in any part there is relaxation, it is only for a moment or two, when the wonted rigidity is again assumed. This is especially true of the eyes. Twice in the nineteen days has he been known to slightly raise his head, and look round as though he were conscious, and then again to pass into the same rigid state. When sustained alone by the head and feet, the thighs slowly *flex* upon the pelvis, and there is a facial expression indicative of pain. The body thus flexed to an angle of forty-five degrees, was laid upon a hard couch upon the floor. The head and chest began very slowly to sink to a horizontal position, and were ten minutes by count in reaching the plane, and this without twitching or tremor, or other evidence of muscular exhaustion, other than the acceleration of the pulse and respiration. Placed in an upright position and left to himself, the body obeys the laws of gravitation without any evidence of volition on the part of the patient. Sustained in an upright position, the legs gradually flex, and the body settles to the floor. When their weight is not sufficient to overcome the rigidity of the muscles—as for instance, the hands and fore-arms—they remain in a given position for an almost indefinite period.

2d. *Color of the Body.* — The patient is usually a little paler than natural, although a blush frequently comes over the surface as in health. The cutaneous capillary circulation is nearly natural; the skin soft and natural; the superficial heat about as in health.

3d. *Temperature.*—The bulb of the thermometer in the mouth, the registry averages 98½; in the rectum, 99.

4th. *Respirations.*—The general average has been from 13 to 16 per minute. When the body was *flexed* and left, head and extremities unsupported, and they were gravitating to a level, the respirations rose to 24 per minute. The respirations are nearly absolutely abdominal, as the elevation of ribs and sternum is imperceptible.

5th. *Circulation.*—The beating of the heart is natural, but rather feebler than should be in health. Sounds all natural. Pulsations average from 65 to 78, sometimes running to 100 when being moved, and at one time to 124 when inhaling ether.

6th. *Nutrition.*—The method of feeding since his entrance to the hospital, has been by inserting an œsophagus tube; and when in position, connecting with a fountain syringe—and by this means, introducing regularly, three times each day, a sufficiency of milk, eggs, and *beef-tea.* There was no evacuation of the bowels after his entrance to the hospital for ten days, when there were free movements of solid fæces of natural color and appearance; and another twelve hours later, since which time, the evacuations have taken place every two days, with some evidence of tenesmus.

7th. *The Urine.*—This has been carefully drawn on different days, so as to ascertain accurately the amount secreted. When not drawn, it is usually voided from three to six times in twenty-four hours, the average amounts varying from twelve to twenty ounces. It has a healthy appearance; contains a little mucus, specific gravity of 1028, and yields neither albumen nor sugar, and is acid in reaction.

8th. *Behavior Under the Influence of Ether.*—The patient has been twice anæsthetized; the first time by Dr. Gradle, who reports a temporary and complete relaxation of the muscles, followed in a few moments by the usual rigidity.

To-day, I caused him to inhale ether for fifteen minutes, with the following results:

At the end of that time, there was partial relaxation of the muscles—thoracic respiration was established, and full, deep inspirations, with elevation of the *ribs* and *sternum*.

The eyes opened as when one awakes from sleep, and were turned to different persons as if fully conscious. He shrank from the threatened snapping of the nose, and performed the only seeming voluntary motion which I have witnessed in the nineteen days, when he raised his hand and removed the napkin which was saturated with ether, and was laid upon his face. His head was slightly raised, and he seemed to look round the room as if *bewildered*. The surface was now flushed and warm; temperature 99 internally. Respirations increased to twenty-four per minute, and the pulse ran up to 124. Inhalation of ammonia and sulphurous acid gas, showed irritation of the nostril. As the etherization passed off, he sunk again, in all respects, into his former state.

*Effect of Electricity.*—The galvano-Faradic current was now tried, as strong as the attendant could well tolerate, with marked *effect*, in contraction of the facial muscles as it was applied over the seventh nerve; so upon the cervical region and the hand. The spinal muscles responded to the application to the cervix and sacrum.

Experiments were continued over different regions of the body, till the fact of response of the muscles to the stimulus of electricity was fully established, but their tonicity was in no wise diminished by its application.

The pupil of the eye also readily contracted under the stimulus of electricity applied to the temples, the same as with a bright light.

Blood flowed moderately freely under the act of wet cupping, and readily coagulated, and had a healthy appearance.

Under the microscope, the disks appear well formed, and the white corpuscles have their relative percentage only.

The patient has now been in this condition about *seventy* days, and what shall be the result, time only can determine. I have thus far sketched what may be termed the gross appearances of what is clearly a case of complicated catalepsy.

Jan. 1st, 1875. P. S.—Since the above report was submitted, the patient has so far recovered the use of his muscular system as to feed himself. The consciousness of what had passed is partial, and the power of sensation becoming again apparent.

---

THE PREVENTION OF ZYMOTIC DISEASES.—Dr. Lyon Playfair, in his address as President of the Health Section of the British Social Science Association, says: "The isolation of patients afflicted with small-pox, scarlatina and measles, will one day become part of hygienic law, though at present it would not be supported by public opinion."

THE ORIGIN OF TRANSFUSION.—According to reliable records, transfusion was first practiced, in 1492, upon the person of Pope Innocent VIII. The operator was a Jewish physician. The experiment was tried three times. The result of the treatment was that the blood-donors, three in number, died, as well as the decrepit pontiff himself.

